# MRI should be routine for all patients with localized prostate cancer? | *Opinion: Yes*

**DOI:** 10.1590/S1677-5538.IBJU.2016.06.03

**Published:** 2016

**Authors:** Ronaldo Hueb Baroni

**Affiliations:** 1 Hospital Israelita Albert Einstein, SP, Brasil

**Keywords:** Magnetic Resonance Imaging, Prostatic Neoplasms, Diagnosis, Watchful Waiting

Magnetic resonance imaging (MRI) has been used for staging prostate cancer (PCa) since the 1990's, more precisely after the advent of the endorectal coil, which enabled significant improvement in the quality of the examination. Also, the standardization of prostate MRI with multiparametric sequences (including high resolution T2-weighted, diffusion and dynamic contrast-enhanced or perfusion images), together with the progressive learning curve by uro-radiologists, contributed to include the method definitively in the list of available procedures for staging prostate cancer (1).

The accuracy of multiparametric MRI (mpMRI) is greater than that of other isolated clinical, laboratory and imaging methods available, with specificities around 85% for detection of extracapsular extension and seminal vesicle invasion (2). Moreover, the incremental value of MRI has been validated around a decade ago in three articles by the interdisciplinary group of Memorial Sloan Kettering Cancer Center, demonstrating that the addition of MRI to the commonly used clinical nomograms significantly increases the accuracy for prediction of organ-confined disease, extracapsular extension and seminal vesicle invasion (3–5).

The indication and acceptance of mpMRI for prostate cancer staging increased after the development and clinical use of 3 Tesla (T) scanners (which have twice the magnet field strength in comparison to more common 1.5 T scanners), allowing the achievement of multi-parametric studies of the prostate without the need for an endorectal coil, with the same resolution and image quality as compared to the studies on 1.5 T scanners with endorectal coil (6).

On the other hand, performing mpMRI for staging of PCa after the biopsy has some limitations. First, a recent meta-analysis with a very large number of studies and patients, evaluating the performance of MRI for local staging of disease, showed high specificities (88-96%) but low sensitivities (57-61%), considering that microscopic infiltration of the capsule or seminal vesicles might be undetectable even with state-of-the-art equipments and protocols (7). Also, there must be a minimum interval of three weeks between prostate biopsy and MRI, to minimize bleeding artifacts that impair the interpretation of the study (8). Finally, it is arguable that very-low and low risk tumors would not benefit from a staging mpMRI, since the chance of extraprostatic disease is small (9).

In this decade, a new and promising application for MRI has emerged: by using standardized interpretation and reporting systems (like PI-RADS and Likert), mpMRI can be used as an additional screening tool to stratify the risk for prostate cancer. mpMRI has the ability to detect small clinically significant tumors in areas that might go undetected on a standardized, random biopsy; on the other hand, clinically insignificant tumors are usually not seen on mpMRI. A recent meta-analysis has shown pooled sensitivities and specificities of 82% for prostate cancer detection in articles that accurately used the PI-RADS criteria (10).

The digital images obtained on mpMRI can be used to improve ultrasound (US) guided biopsy procedures by using MRI-US fusion techniques, whether cognitive (when the radiologist or urologist visually defines the region of the suspected lesion on mpMR images and directs additional fragments to this region) or real-time (when the digital mpMR images are uploaded in the US equipment and allow real-time visualization of the concordant MR and US images during the biopsy). Many recent articles have demonstrated that, in comparison to random biopsy, fusion biopsy techniques improve in up to 30% the detection of clinically significant PCa (reducing disease morbidity and mortality), while decreasing the detection of insignificant disease (reducing overtreatment) (11, 12).

**Figure 1:**
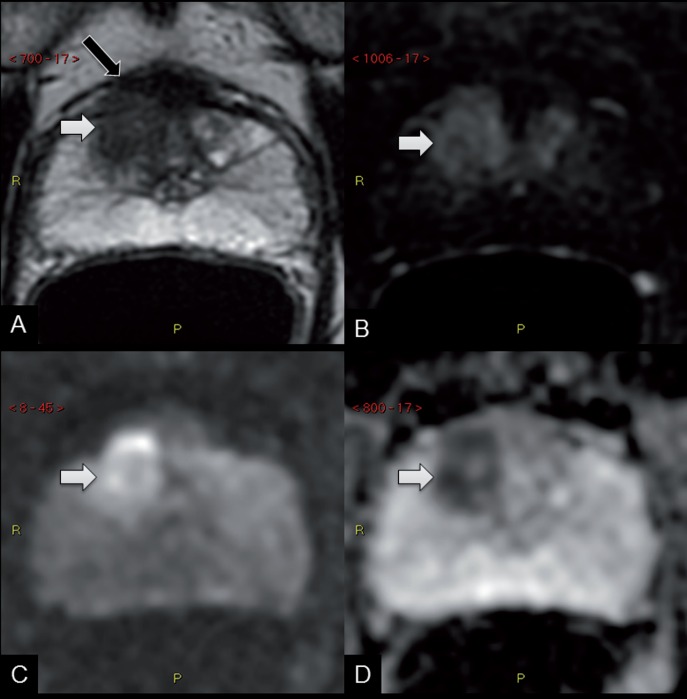
59 years old man, elevated PSA (8 ng/mL), no prior biopsy. mpMRI performed on a 3 Tesla scanner without an endorectal coil revealed a focal lesion of the right anterior transition zone of the prostate (white arrows), with homogeneous low signal intensity on the T2-weighted images (A), early enhancement on the dynamic post-contrast sequence (B), marked restricted diffusion on the diffusion-weighted (c) and ADC sequences (D), and suggestive signs of infiltration of the fibromuscular stroma and extension to the periprostatic fat (black arrow), highly suspicious for prostate cancer (PI-RADs 5). Real-time MR/us fusion biopsy confimed a Gleason 7 (4+3) tumor in this area, and prostatectomy revealed a Gleason 8 (4+4) pT3a lesion.

By performing mpMRI prior to biopsy, a paradigm-shift is evolving, since lesions highly suspicious for clinically significant tumors are been locally staged by mpMRI before histological confirmation of the disease. This may sound unorthodox, given the historical algorithm of prostate cancer detection and staging, but is the usual workflow in many other tumors (such as kidney cancer), and might increases the capability of the radiologist to locally stage the disease in prostates without biopsy-related artifacts (Figure-1).

Although mpMRI has been currently used for PCa detection in many centers, the definitive incorporation of mpMRI in the screening algorithm of PCa in the general male population is still restricted by relevant issues: the exam is costly and time-consuming, the availability of 3-Tesla scanners is limited, and the need of contrast-injection for the perfusion part of the protocol carries the (small) risk of allergic reactions and other rare side-effects. Therefore, many center are investing on faster, cheaper, non-invasive and harmless MR protocols for PCa screening, generically called bi-parametric MRI (since it includes only T2 and diffusion-weighted sequences), which can perform on more widely available 1.5 Tesla scanners without an endorectal coil. Two recent articles demonstrated a good accuracy of bi-parametric MRI, associated with serum-PSA levels, for the detection of PCa in correlation to biopsy (13, 14).

In conclusion, mpMRI has already been accepted as a valuable method for local staging in patients with intermediate to high-risk PCa. However, given the growing applicability of mpMRI for the screening of clinically significant tumors (and supported by some urologists' perspectives on prostate cancer imaging (15)), I would humbly suggest a rephrasing of the proposed theme of this article: in the near future, all men with suspicion for prostate cancer should undergo MRI.
